# Metabolic Reprogramming of Immune Cells at the Maternal-Fetal Interface and the Development of Techniques for Immunometabolism

**DOI:** 10.3389/fimmu.2021.717014

**Published:** 2021-09-09

**Authors:** Yiqiu Wei, Jinli Ding, Jianan Li, Songchen Cai, Su Liu, Ling Hong, Tailang Yin, Yan Zhang, Lianghui Diao

**Affiliations:** ^1^Reproductive Medicine Center, Renmin Hospital of Wuhan University, Wuhan, China; ^2^Shenzhen Key Laboratory for Reproductive Immunology of Peri-Implantation, Shenzhen Zhongshan Institute for Reproduction and Genetics, Shenzhen Zhongshan Urology Hospital, Shenzhen, China; ^3^Shenzhen Jinxin Medical Technology Innovation Center, Co., Ltd., Shenzhen, China; ^4^Department of Clinical Laboratory, Renmin Hospital of Wuhan University, Wuhan, China

**Keywords:** maternal-fetal tolerance, metabolomics, cell metabolism analysis, reproductive immunology, natural killer cells, macrophages, T cells

## Abstract

Immunity and metabolism are interdependent and coordinated, which are the core mechanisms for the body to maintain homeostasis. In tumor immunology research, immunometabolism has been a research hotspot and has achieved groundbreaking changes in recent years. However, in the field of maternal-fetal medicine, research on immunometabolism is still lagging. Reports directly investigating the roles of immunometabolism in the endometrial microenvironment and regulation of maternal-fetal immune tolerance are relatively few. This review highlights the leading techniques used to study immunometabolism and their development, the immune cells at the maternal-fetal interface and their metabolic features required for the implementation of their functions, explores the interaction between immunometabolism and pregnancy regulation based on little evidence and clues, and attempts to propose some new research directions and perspectives.

## Introduction

Classical reproductive immunology considers that pregnancy can be understood as a model for “allograft transplantation.” The establishment of a normal pregnancy depends on maternal immune recognition and tolerance to the embryonic alloantigens (1). The interaction between immune cells and cytokines at the maternal-fetal interface creates an immune tolerance microenvironment, preventing the fetus from the maternal immune system attacks and maintaining a certain degree of immunity to protect the mother from harmful pathogens (2, 3). The immune microenvironment at the maternal-fetal interface is mainly composed of decidual stromal cells (DSC), decidual epithelial cells (DEC), trophoblast cells, decidua immune cells (DIC), and the soluble factors. The establishment of pregnancy induces a series of maternal immune-inflammatory responses and adaptive changes in material and energy metabolism (4). With the progress of pregnancy, the maternal-fetal interface immune microenvironment shows a dynamic equilibrium: during early pregnancy, the maternal-fetal interface changes from an inflammatory microenvironment that is conducive to embryo implantation and pregnancy establishment to an immune tolerance microenvironment that is conducive to pregnancy maintenance and fetal growth and development. Until late pregnancy, it gradually tends to inflammatory transition, and is endogenously prepared for delivery. When the regulation of maternal-fetal immune tolerance is disturbed, the result can be an excessive immune response to the fetus and the development of immune rejection, leading to pathological pregnancy or even pregnancy loss.

Immune cell metabolic reprogramming is a general term for the response of immune cells to crucial changes in the environment, involving changes in critical metabolic enzymes, metabolites, and metabolic pathways. Various cytokines and antigens can regulate the metabolism of immune cells. Then, metabolic reprogramming occurs and affects the outcome of the immune response. The metabolic level of the cell itself is also closely related to its function (5–7). Many immune cells are infiltrated in the uterus, and the success of pregnancy immunity depends to a certain extent on their crosstalk with trophoblast cells (8). Immune cells, such as natural killer (NK) cells, macrophages (Mφ), T cells and dendritic cells (DC), exhibit specific phenotypes and functions through a series of cell differentiation and participate in the regulation of reproductive processes (9). In addition to meeting the material basis required for the growth and development of the fetus, metabolic reprogramming also plays a role in regulating the immune state. Finally, it establishes an “immune tolerance” microenvironment in the uterus, achieving “peaceful coexistence” between mother and fetus (10). However, the differentiation and regulatory mechanism of endometrial immune cells remain to be elucidated. Whether immunometabolism regulates the phenotype and function of immune cells and regulates the physiological processes of reproduction remains further revealed. This review focuses on the leading techniques used to study immunometabolism and their development, summarizes the functions of immune cells at the maternal-fetal interface, and explores the potential role of immune cell metabolic reprogramming in the maternal-fetal interface to provide new ideas for the diagnosis and treatment of clinical pregnancy-related diseases.

## Leading Techniques for Detection of Immune Cell Metabolism

**Figure 1:**
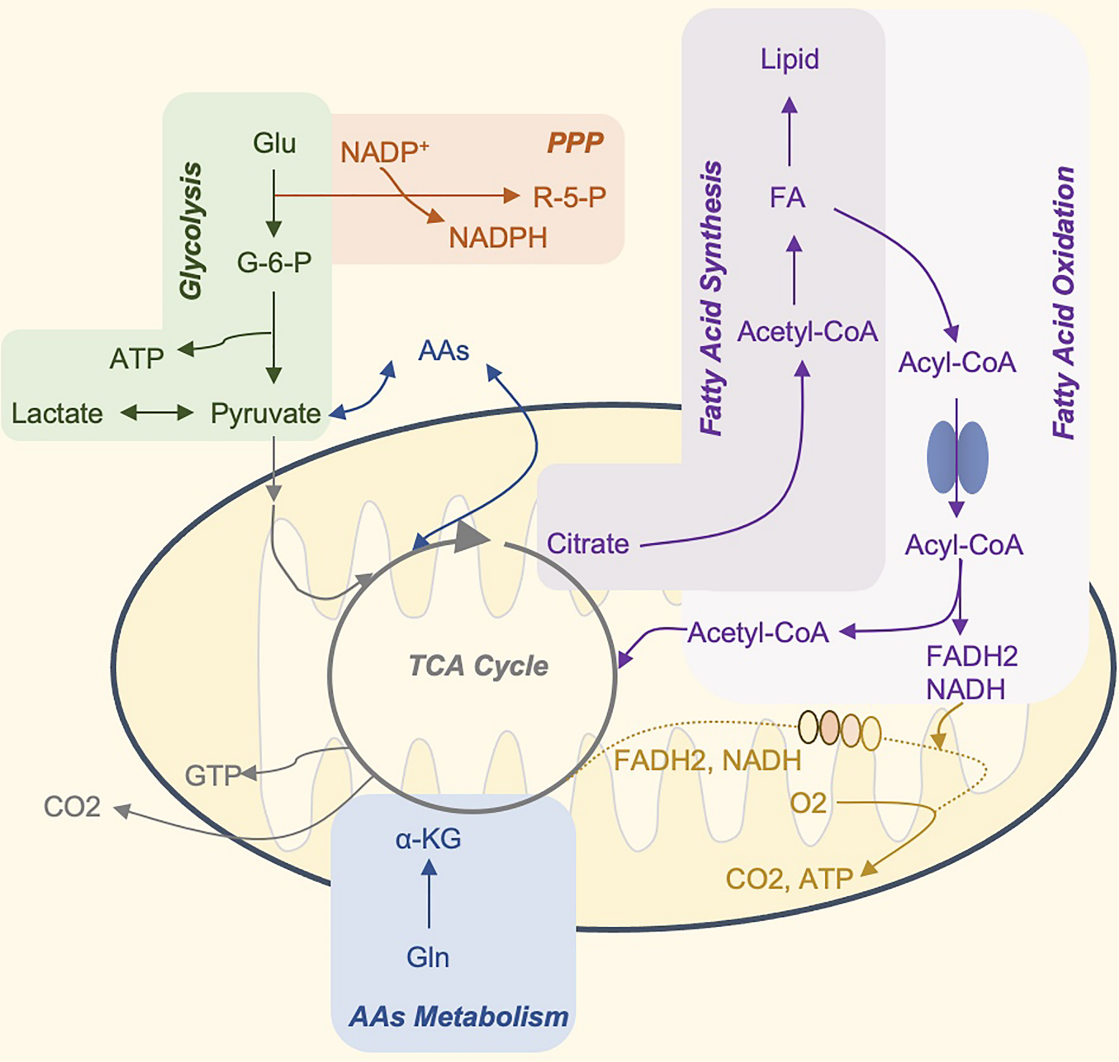
The Core Metabolic Pathways of Immune Cells. Glucose is the primary energy supply substance. It can produce ATP through glycolysis, or tricarboxylic acid cycle, and oxidative phosphorylation. In addition, glucose 6-phosphate, the product of the first step of glycolysis, can participate in the production of pentose phosphate and NADPH by PPP, and participate in nucleotide metabolism and glutathione reduction, respectively (not shown in the figure). The TCA cycle is the hub of sugar, fat, and amino acid metabolism, and the three major nutrients can be transformed into each other through this cycle. Citrate can be used as a carrier to transport acetyl-CoA to the cytoplasm. Acetyl-CoA is the raw material for the synthesis of fatty acids and cholesterol. Many amino acids can be synthesized from intermediate products of the TCA cycle, and amino acids and fatty acids can also be used as alternative substrates for the process. For example, acetyl-CoA produced by β-oxidation of fatty acids and α-ketoglutarate produced by the deamination of glutamate, can enter the TCA cycle and drive OXPHOS. Glu, glucose; G6P, glucose 6-phosphate; OXPHOS, oxidative phosphorylation; ATP, adenosine triphosphate; GTP, guanosine triphosphate; PPP, pentose phosphate pathway; AAs, amino acid synthesis; FA, fatty acids; R-5-P, ribose 5-phosphate; NADPH, reduced form of nicotinamide adenine dinucleotide phosphate; NADH, nicotinamide adenine dinucleotide FADH2, reduced flavine adenine dinucleotide.

Metabolism is a crucial physiological activity in cells. There are six main metabolic pathways in immune cells, including glycolysis, tricarboxylic acid (TCA) cycle, pentose phosphate pathway (PPP), fatty acid oxidation (FAO), fatty acid synthesis (FAS), and amino acid metabolism (10). Their interaction provides energy and nutrients for the maintenance of cell life activities (**Figure 1**). Cell metabolism is more than just a process of adenosine triphosphate (ATP) production, biosynthesis, and catabolism. Metabolites and metabolic fluxes can regulate cellular signaling pathways and post-translational modifications (PTM) (11), leading to changes in gene expression and even in the epigenome. For example, post-translational histone modification can be affected by changing local concentrations of key metabolites, thereby regulating transcription and other DNA-templated functions (12). Metabolic activity can regulate cell apoptosis and autophagy (13, 14), and metabolic enzymes can also act as cellular RNA binding proteins to participate in the post-transcriptional control of specific mRNAs (15, 16). Finally, metabolites can be directly used as signal molecules to affect pro- and anti-inflammatory results (17, 18).

In the past few decades, people have been developing tools to detect changes in cellular metabolism. Traditional bulk metabolism research methods include extracellular flux analysis (EFA), steady-state metabolomics, etc. With the advent of single-cell applications, researchers can now estimate the metabolic status of individual immune cells in clinical samples. Technological advancements in measuring metabolites and metabolic activity have provided researchers with more tools than ever to understand and test the complexity of immunometabolism. Here is a brief introduction to the leading techniques used to study immunometabolism and its developmental history.

The EFA technique was initially used to study the effects of drugs *in vitro* by measuring cellular respiration and acidification. Researchers have developed various tools for its development, but the efficiency is still low, and the application is relatively limited (19). Since introducing the Seahorse Bioscience XFe Extracellular Flux Analyzer in 2009, it has brought innovation to this technology. It is the first in the world to use 24-well or 96-well microwell plates as a platform, which can record the extracellular acidification rate (ECAR) and oxygen consumption rate (OCR) in real-time as key metabolic readings and provide indirect measurements of glycolysis and mitochondria respiration (20). It dramatically expands the research horizon of cell metabolism and extends it to the field of immune cells. It provides tools for exploring the upstream biology and cellular and molecular mechanisms that control immune cell responses through real-time metabolic analysis of live cells. Since 2012, there have been studies using this method to study the metabolic changes of dendritic cells (21), T cells (22), monocytes (23), and macrophages (24). However, it cannot provide detailed information about the metabolic pathways other than glycolysis and the TCA cycle in the mitochondrial activity. It also cannot capture a single cell’s metabolism information.

Flow cytometry (FCM) is one of the most straightforward and informative single-cell methods for analyzing metabolic characteristics. In 1969, Van Dilla Fulwyler et al. invented the first fluorescence detection cytometer to quickly count human white blood cells (25). With the monoclonal antibody technology proposed by Kochler and Milstein in 1975 (26), the combination of cytometer and immunity and continuous development have gradually formed the current flow cytometer. It is no longer only used for cell counting but quantifies proteins, nucleic acids, and metabolites. Since this technique has been developed for many years, commercially metabolite fluorescent dyes and analogs are abundant and easy to use. Immune cells incubated with a fluorescently labeled substrate will occupy the substrate with an endogenous transporter. After washing the exogenous matrix, flow cytometry can quantify the uptake of metabolites from the matrix by the cells. The additional advantage of these techniques is that they required only a small number of cells, making flow cytometry a popular choice for rapidly verifying differences in metabolic activity (27, 28).

Cytometry + time of flight (CyTOF) is a flow cytometry technique based on heavy metal isotope-labeled antibodies. The key feature is that it no longer needs to use fluorescein-labeled antibodies like traditional flow cytometry. Since the sample will enter the mass spectrometer for analysis, it is also called mass spectrometry flow cytometry (29, 30). The application of this technique, on the one hand, has dramatically increased the number of flow detection channels to hundreds, increasing the amount of information obtained from a single sample. On the other hand, because the detection overlaps and the background are very low in cell analysis (since heavy metals will not naturally appear in the cell), avoiding signal interference between channels and improving data reliability. CyTOF is an excellent tool for tumor immunology. It can be used to phenotype tumor infiltrating immune cells and understand the metabolic spectrum of immune cells in the tumor microenvironment (31). CyTOF is also used to analyze histone acetylation markers and cell lineage markers simultaneously, thereby enabling the detection of histone acetylation changes in samples (32). Therefore, CyTOF that combines acetylation markers, metabolic signals, and lineage markers can finally reveal many new connections between metabolism and epigenetic regulation in complex immunocytes.

Although combined with mass spectrometry, the label’s quantity of detectable metabolites is limited because CyTOF is an antibody labeling-based technique. Moreover, the three metabolic detection techniques mentioned above are all indirect, which cannot directly measure the metabolites’ content in cells. So, is there a way to directly measure the level of metabolites? The answer is metabolomics.

Metabolomics is a new emerging omics after genomics, proteomics, and transcriptomics. Since 1998, related research has increased rapidly, but it still lags far behind genomics and proteomics (33). For a given time point, metabolomics analysis can measure the steady-state levels of a series of metabolites through a liquid or gas chromatography-mass spectrometry (LC-MS or GC-MS) and nuclear magnetic resonance (NMR) (34–36). Most MS-based methods still require high levels of input flux (approximately 100,000 s of cells). With technology development, single-cell metabolomics tools have been gradually developed and applied (37). Representative single-cell metabolism mass spectrometry tools include Matrix-Assisted Laser Desorption Ionization Time of Flight (MALDI-TOF) (38, 39), Secondary Ion Mass Spectrometry (SIMS) (40), Direct Infusion Mass Spectrometry (DI-MS) (41), Capillary Electrophoresis Mass Spectrometry (CE-MS) (42), etc. Metabolomics analysis of single-cell expands its applications. Metabolomics can detect metabolite content in all metabolic pathways, identify changed metabolite levels, and provide information for studying the role of metabolites in regulating cell function (43). Metabolomics analysis can be divided into untargeted and targeted metabolomics. Untargeted metabolomics analysis can detect as many metabolites as possible, but it requires expertise in metabolite identification, and its sensitivity may also be limited. In other words, although thousands of signals can be detected, only a few substances can be identified. However, this can lead to discoveries and is very suitable for the development of biomarkers. Targeted metabolomics evaluates pre-selected metabolites, generates data with higher sensitivity, and allows precise absolute quantification of metabolite concentrations, facilitating the analysis of known changes in metabolite levels (44, 45).

Nowadays, we can use various metabolic detection techniques to observe the processes of metabolic remodeling in different immune cell subsets. These techniques have their advantages and disadvantages (**Table 1**). However, the raw data obtained by these techniques are either indirect or covered limited. In order to promote the progress of these techniques and the next generation of immunometabolism research, there are two main directions: 1) Improve the depth and quality of data analysis of single-cell level analysis; 2) Improve ability to analyze the temporal and spatial distribution of metabolism.

**Table 1:** Leading techniques for detection of immune cell metabolism and their advantages and disadvantages.

Techniques	Advantages	Disadvantages	Time of appearance in immunology	Representative works
Extracel-lular flux analysis	Simple Affordable Fast	Unable to detect single cell metabolism Only the information of glycolysis and TCA cycle in mitochondria can be obtained Indirect detection	1970s	Katherine et al. 2016 (46)
Flow cytometry	Common laboratory instruments Affordable Fast	Indirect detection Low throughput Fluorescence may cause interference to the background	1980s	Argüello et al. 2020 (47)
CyTOF	High throughput No interference between channels Fast	Indirect detection Expensive equipment	2010s	Horowitz et al. 2013 (48)
Steady state metabolo-mics	Direct detection It can target or untargeted detect the entire metabolome	Expensive equipment Metabolome is unstable and easily affected by storage conditions High-level technical personnel need	2010s	Tannahill et al. 2013 (49)
Single cell metabolo-mics	Direct detection It can target or untargeted detect the entire metabolome	Expensive equipment High-level technical personnel need Low abundance and low ionization efficiency metabolites cannot be detected	2010s	Hartmann et al. 2021 (50)

Based on the above detection techniques, researchers have promoted the understanding of immunometabolic reprogramming. Next, this review discusses the function of immune cells at the maternal-fetal interface and their metabolic preferences required to perform this function explores the potential role of immune cell metabolic reprogramming in the maternal-fetal immune interface.

## The Metabolic Reprogramming of the NK Cells in the Endometrium and Decidua

NK cells are the most abundant immune cells in the endometrium during embryo implantation and placenta formation. In early pregnancy, decidual NK cells (dNK cells) account for about 50% to 90% of decidual lymphocytes, and it gradually decreases during the middle and late pregnancy (51). They play a crucial role in the uterine immunity to promote blood vessel remodeling at the maternal-fetal interface, maintain the stability of the immune microenvironment, enable the healthy growth of the fetus, and protect the mother from harmful pathogens through appropriate inflammation (52, 53).

Decidual NK cells are mainly CD56^bright^CD16^-^ NK cell subgroups. Unlike the cytotoxic CD56^dim^CD16^+^ NK cells in peripheral blood, it is a highly immunomodulatory cell (54) that can secrete cytokines such as interferon-γ (IFN- γ) promotes angiogenesis and tissue remodeling (55). The function of dNK cells in early pregnancy is regulated by the expression of trophoblast human leucocyte antigen-I (HLA-I) (such as HLA-C/G/E) and the interaction of inhibitory or activating receptors (such as KIR, CD94/NKG2, ILT family) with trophoblast (56). A large amount of evidence indicates that the specific combination of highly polymorphic killer cell immunoglobulin-like receptors (KIR) on dNK cells and HLA-C on invasive trophoblasts affect decidual vascular recasting and thus affects the success of pregnancy (57).

**Figure 2:**
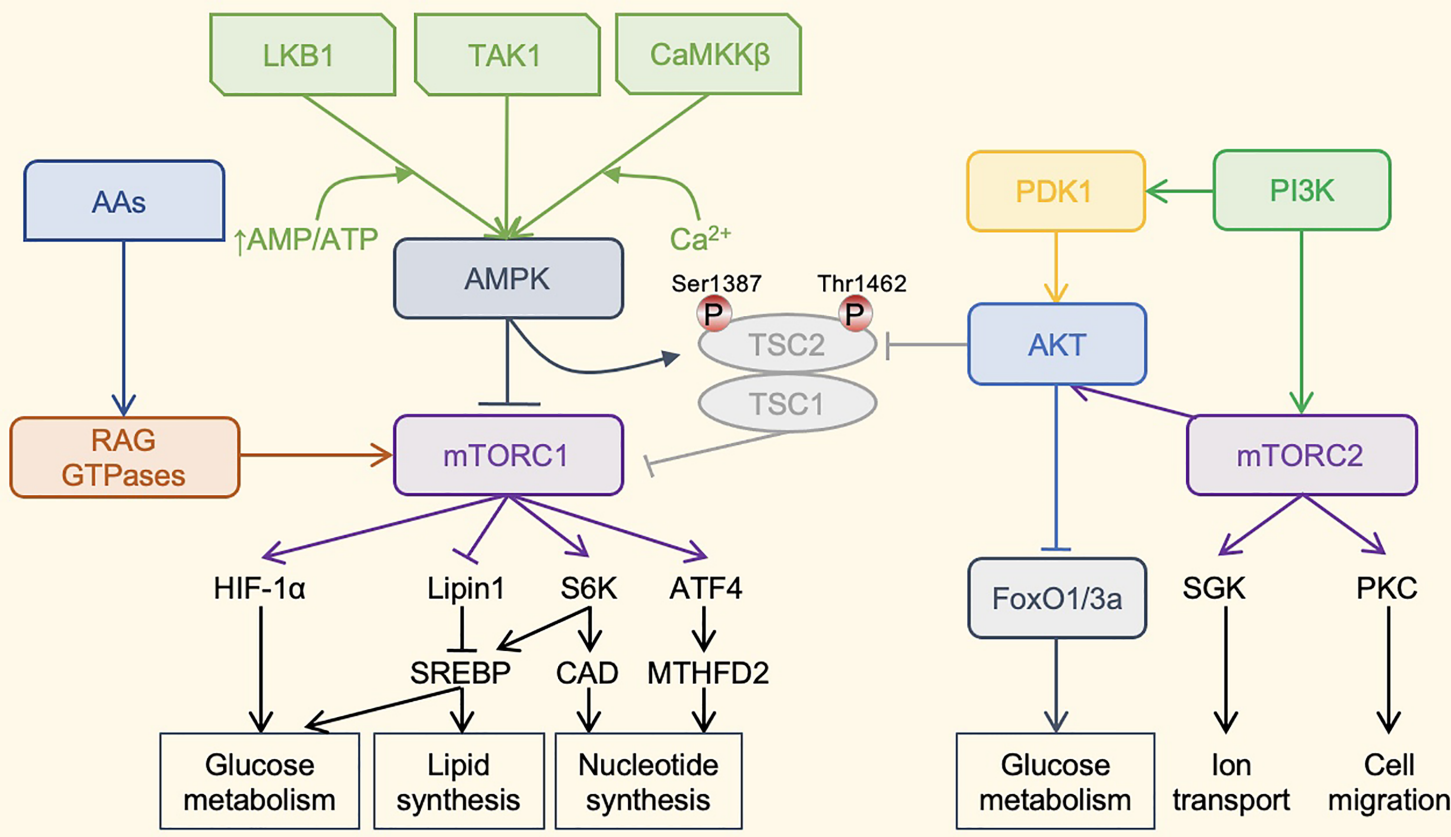
The mTOR-centered Metabolic Regulatory Signaling Pathway. mTOR is a crucial eukaryotic cell regulator, which affects the expression of cytokines in cells, participates in immunosuppression, and regulates cell growth. mTOR contains two complexes. mTORC1 promotes cell growth by promoting glucose metabolism. It can enhance the translation of the transcription factor HIF-1α, thereby promoting glucose metabolism. It can also activate the transcription factor SREBP to promote lipid synthesis and meet the formation of membranes during cell growth. mTORC1 also promotes the synthesis of nucleotides required for DNA replication and ribosome synthesis during cell growth and proliferation. mTORC1 promotes the expression of ATF4, further increases the expression of MTHFD2, and provides carbon units for purine synthesis. Phosphorylation of S6K1 can activate the CAD enzyme and promote pyrimidine synthesis. mTORC1 is mainly regulated by upstream AMPK. AMPKa is activated by phosphorylation by LKB1, TAK1, or CaMKKb kinase. LKB1 promotes the enhancement of AMPK phosphorylation under a high AMP/ATP ratio. Activated AMPK can cause AMPK-dependent phosphorylation of Ser 1387 of TSC2 and reduce mTORC1 activation. AMPK can also directly phosphorylate Raptor to reduce the activity of mTORC1. The TSC1–TSC2 complex also integrates the PI3K–Akt pathway, which regulates the activity of mTORC1 through the AKT-dependent phosphorylation of Thr1462. In addition, amino acids can activate mTORC1 through the RAG family of small GTPases. The most important role of mTORC2 may be the phosphorylation and activation of Akt. Akt is a key effector of insulin/PI3K signaling. Once activated, Akt promotes cell survival, proliferation and growth through phosphorylation and inhibition of several key substrates, including FoxO1/3a transcription factor and TSC2. Finally, mTORC2 also phosphorylates and activates SGK1 and PKC, and regulates ion transport and cell migration. PI3K can directly activate mTORC2 through phosphorylation. mTOR, mammalian target of rapamycin; HIF-1α, hypoxia-inducible factor-1α; SREBP, Sterol regulatory element binding proteins; ATF4, activating transcription factor 4; MTHFD2, methylenetetrahydrofolate dehydrogenase 2; S6K1, ribosomal protein S6 kinase 1, CAD, carbamoyl-phosphate synthetase 2, aspartate transcarbamoylase, dihydroorotase; AMPK, adenosine 5’-monophosphate-activated protein kinase; LKB1, liver kinase B1; TAK1, transforming growth factor beta-activated kinase 1; CaMKKb, Ca^2+^/calmodulin-dependent protein kinase kinase beta; TSC, tuberous sclerosis complex; PI3K, phosphoinositide 3-kinase; Akt, serine-threonine kinase; PDK1, phosphoinositide dependent kinase-1; RAG GTPases, ras-related guanosine 5´-triphosphate-binding protein; FoxO1/3a, forkhead box protein O1/3a; SGK1, serum/glucocorticoid-regulated kinase 1; PKC, protein kinase C; Thr1462, threonine 1462; Ser 1387, serine 1387.

The metabolic regulation of NK cells is driven by complex molecular mechanisms, among which the mammalian target of rapamycin (mTOR) signaling pathway is the most important and the most widely studied in metabolic regulation (**Figure 2**). During NK cell activation, glycolysis and mitochondrial function are enhanced *via* the activation of mTOR. The pharmacological inhibition of mTOR by rapamycin reduces the up-regulation of IL-2/IL-12-stimulated glycolysis in mouse NK cells and decreases IL-2-stimulated levels of human NK cell glycolysis (58, 59). Compared with CD56^dim^ NK cells, CD56^bright^ NK cells show a more robust metabolic response to IL-2 or IL-12/IL-15 stimulation and have higher mTOR activity. It preferentially upregulates the nutrient receptors CD71 and CD98 in an mTOR-dependent manner, expressing higher levels of glucose transporter 1 (GLUT1) to absorb glucose quickly (60). However, excessive activation of mTOR may cause mitochondrial fragmentation, thereby impairing mitochondrial function (61).

Although lipid synthesis is essential for the metabolic reprogramming of T cells, inhibition of the lipid synthesis pathway has the most negligible impact on NK cell effector function and proliferation (62, 63). It is still unknown whether fatty acids (FA) can provide energy for NK cells as an essential nutrient. FA administration, and peroxisome proliferator-activated receptor (PPAR) agonists, inhibited mTOR-mediated glycolysis and inhibited NK cell effector function and metabolism (64). Metabolic activation is essential for NK cells to produce effector proteins (including IFN-γ and Granzyme B) and to form correct immune synapses with target cells (59, 64).

Glutamine (Gln) is another crucial substrate for cell metabolism; it enters the TCA cycle but does not sustain oxidative phosphorylation (OXPHOS) in activated NK cells. It plays an essential role for amino acid-controlled c-Myc for NK cell metabolism and function (65). Gln helps absorb the amino acids necessary for the high rate of c-Myc protein synthesis through the L-type amino acid transporter SLC7A5, counteracting the continuous degradation of c-Myc, while Gln withdrawal results in the loss of c-Myc protein. c-Myc controls the expression of metabolic mechanisms, including glucose transporters and glycolytic enzymes, which are necessary to support increased metabolism during NK cell activation (65).

However, the current research mainly focuses on peripheral blood NK cells, and the research on dNK cell metabolism is relatively limited. Since dNK cells are the most abundant immune cells in the maternal-fetal interface, studying their metabolic reprogramming is likely to yield breakthrough achievements.

## Metabolic Reprogramming of Macrophages Affects Polarization and Plays a Role in the Maternal-Fetal Interface

Macrophages are the second largest immune cells population in the endometrium and decidua after NK cells, accounting for 20-30% of lymphocytes (66). Generally, macrophages are classified into pro-inflammatory M1 macrophages and anti-inflammatory M2 macrophages based on their activation status (67). The “plasticity” of macrophages allows them to respond rapidly to the immune response triggered by embryonic implantation, which plays a pivotal role in immune regulation and promotes angiogenesis (4). During pregnancy, the number and ratio of M1/M2 macrophages at the maternal-fetal interface change dynamically: In the early stage of embryo implantation, decidual macrophages are dominated by M1 macrophages, and then, metabolic reprogramming occurs, more macrophages are polarized into M2 macrophages, which prevents rejection of the fetus. Until late pregnancy, the polarization of macrophages gradually tends to change to inflammation, which is endogenously prepared for delivery (68, 69). The dynamic balance between M1 and M2 macrophages is essential for the whole process of normal pregnancy (70). Inappropriate macrophage polarization, whenever it occurs, is usually associated with abnormal pregnancy, such as spontaneous abortion (71), preterm delivery (PTL) (72), preeclampsia (PE) (73), fetal growth restriction (FGR) (74).

Macrophages with different polarization states have apparent differences in glycometabolism pathways. Pro-inflammatory M1 macrophages are mainly powered by anaerobic glycolysis, providing instantaneous and rapid energy to eliminate microorganisms that invade the body, while mitochondrial OXPHOS continuous generates energy for anti-inflammatory M2 macrophages (75). Studies have shown that a normal mitochondrial function is required for the polarization/repolarization of macrophages to M2 macrophages (76). When lipopolysaccharide (LPS) and IFN-γ are stimulated, the mitochondrial function of macrophages is impaired or inhibited, the level of OXPHOS is reduced, and anaerobic glycolysis has increased the polarization of macrophages to M1 type. Decreased OXPHOS leads to the accumulation of TCA cycle metabolites such as succinate, and upregulates hypoxia-inducible factor-1α (HIF-1α) (49). HIF-1α can induce the transcription of related transcription factors, such as nuclear factor κB (NF-κB), and positively regulate related key enzymes (such as hexokinase) and transporters (such as GLUT1) to promote metabolic reprogramming of anaerobic glycolysis and PPP, thereby affecting the polarization and biological functions of macrophages (5).

In recent years, many researchers have tried to use glycometabolism reprogramming to regulate the polarization of macrophages and change their functions as a means of disease prevention and treatment. Wenes M et al. have reported that blocking the regulation in development and DNA damage responses 1 (REDD1) of macrophages can change their metabolism and stimulate their glycolysis. Promote macrophages and tumor cells to start “glucose competition”, so that a common and robust blood vessel barrier is formed around the tumor, blocking the metastasis of cancer cells (77). This suggests that glycometabolism reprogramming can change the polarization state of macrophages and affect their related biological functions. Van den Bossche J et al. have also found that restoring mitochondrial function through inducible nitric oxide synthase (iNOS) inhibition may help improve the reprogramming of M1 to M2, thereby controlling inflammation (76). Herein, glycometabolism reprogramming to change the function of macrophages may be used as a therapeutic target for diseases.

About lipid metabolism, researchers have observed different levels of arachidonic acid mobilization and other FA changes in different differentiated types of macrophages, which indicates that the lipidome can reflect the process of cell membrane remodeling and macrophage polarization (78). In the polarization of M2 macrophages stimulated by IL-4, FAs absorption and FAO levels increased significantly. This is mainly due to the activation of the transcription factor recombinant signal transducer and activator of transcription 6 (STAT6) by IL-4, which in turn induces the synthesis of peroxisome proliferative actives receptor-γ coactivator-1β (PGC-1β) (79). At the transcriptional level, peroxisome proliferator-activated receptor γ (PPARγ) is a key transcriptional regulator of macrophage mitochondrial function and FAO, and can enhance alternative activation (49, 80). Lipids can also regulate protein functions by acting as remote signaling molecules. For example, the enhanced esterification of cholesterol acyl transferase 1 (ACAT1) induces more free cholesterol accumulation, and causes the up-regulation of inflammatory signals like toll-like receptors (TLR) and NF-κB signals through lipid rafts (5). This signal is also a key factor in the phosphatidylinositol 3-kinase (PI3K)/serine-threonine kinase (Akt) signal pathway. Its activation can lead to increased polarization and migration of M1 macrophages and promote the secretion of inflammatory cytokines (81).

OXPHOS and amino acid metabolism are also closely related to M2 polarization and anti-inflammatory properties. Inhibiting the level of OXPHOS can reduce the expression level of arginase 1, which is harmful to tissue repair and remodeling (82). Gln deprivation also reduces the polarization of M2 (83). Gln, like glucose, is considered an important energy source for immune cells. Xiao W et al. have reported that Gln supplementation in the diet of rats can reverse the impaired function of macrophages to some extent (84). On the contrary, inhibition of glutamine synthetase can promote the metabolic reprogramming of M2 macrophages to polarize to M1 and promote inflammation.

In conclusion, the dynamic homeostasis and function of macrophages during pregnancy are influenced by various metabolites in the body, and in turn, their metabolic reprogramming plays a crucial regulatory role in maintaining the microenvironment at the maternal-fetal interface.

## The Influence of T Cell Metabolism Reprogramming on the Establishment of the Maternal-Fetal Interface

There are many subsets of T cells, which play different roles in establishing and maintaining pregnancy. Decidual T cells account for about 10% to 20% of decidual lymphocytes (85). Compared with peripheral blood, the proportion of CD4^+^ and CD8^+^ T cells in decidua is inverted, with CD8^+^ T cells predominantly (45%~75%) (86). As the pregnancy progresses, the number of T cells in the maternal-fetal interface gradually increases. T cells become the dominant lymphocyte in late pregnancy, accounting for about 60% of the decidual lymphocytes (87). CD4^+^ T cell subsets include helper T cells (Th) and regulatory T cells (Treg), and Th cells can be divided into Th1, Th2 and Th17 (88, 89). They regulate immunity by secreting different cytokines.

Th1/Th2 cell-mediated immune balance is one of the primary mechanisms for maintaining maternal-fetal immune tolerance. Under normal pregnancy conditions, the Th1/Th2 balance is biased towards Th2, because Th2-type cytokines (IL-4, IL-5, IL-9 and IL-13) can inhibit inflammation, reduce tissue damage, and stimulate growth and invasion of trophoblast cells, and increase uterine receptivity (86). Once this Th2-type immunodominance is broken, the incidence of spontaneous abortion increases significantly (90).

Th17/Treg is another independent cytokine network following Th1/Th2. It has been reported that both Th17 and Treg are differentiated from initial CD4^+^ T cells in a transforming growth factor-β (TGF-β) concentration-dependent manner, driven by high concentrations of TGF-β initial CD4^+^ T cells differentiate into Tregs, and at low concentrations differentiate into Th17 (91). It has also been reported that decidual Treg cells are selectively recruited from the peripheral blood to the maternal-fetal interface through the action of chemokines CCL2 (92), CCL19 (93), CXCL12 (94), and chemokine receptors CCR5 (95), CCR7 (96) and CXCR4 (94). Treg cells in the maternal-fetal interface of normal pregnancy are significantly higher than those in the peripheral blood and the decidua of patients with spontaneous abortion (97). The Th17 cells and IL-17 secreted by Treg cells jointly promote the proliferation of trophoblast cells and invade the decidual tissue, which plays a positive role in the immune microenvironment of the maternal-fetal interface (98). The abundant Treg at the maternal-fetal interface in the early pregnancy also inhibits the hyperproliferation of Th17 and reduces the production of IL-17 through paracrine or direct contact. Once the number of Treg cells is reduced, or the function is impaired, the excessive enrichment of Th17 will cause excessive inflammation, which will cause a variety of adverse pregnancy outcomes, such as unexplained recurrent spontaneous abortion (URSA) and PE (99, 100).

Decidual CD8^+^ T cells also play an essential role in establishing the maternal-fetal interface (87). The function of CD8^+^ T cells in the endometrium of ordinary non-pregnant women is similar to that of peripheral blood CD8^+^ T cells. It has potent cytotoxicity and exerts immune protection function by releasing cytotoxic particles. During pregnancy, decidual CD8^+^ T cells exhibit temporary dysfunction, with high expression of IFN-γ, low expression of perforin and granzyme B (101, 102). Trophoblasts can promote the expression of CTLA-4 and Tim-3 molecules on decidual CD8^+^ T cells in an HLA-C/G-dependent manner. Decidual CD8^+^ T cells co-expressing Tim-3, CTLA-4, or PD-1 are highly proliferative, can secrete large amounts of Th2 cytokines and play an active role in maintaining maternal-fetal tolerance (102–104).

T cells display different metabolic profiles according to their activation status. Naive T cells mainly rely on OXPHOS to maintain a resting state, while activated T cells maintain their growth by switching to glucose and lipid metabolism. The traditional view is that the aerobic glycolysis rate of proliferating cells is very high, even if there is enough oxygen to support OXPHOS. This phenomenon is called the Warburg effect (105). Substrate-level phosphorylation produces ATP during glycolysis, pyruvate is converted to lactate, and biological macromolecules are synthesized for cell growth and division (106). These metabolic changes can be directly triggered by T cell receptors (TCR). In naive T cells, costimulatory molecules CD28 cooperate with the TCR of antigen-presenting cells (APC) to upregulate the membrane expression of the GLUT1 through the mTOR pathway to promote glucose absorption (107, 108). These effects can be offset by immunosuppressive molecules, including CTLA4 and PD-1, which prevent the immune response by inhibiting glucose uptake and promoting endogenous fatty acid oxidation (109).

Th1, Th2 and Th17 show strong glycolysis preferences in mitochondrial metabolism, while Treg cells exhibit mixed metabolism involving glycolysis, lipid oxidation, and OXPHOS (110). In particular, Th17 cells have an increased dependence on glycolysis. HIF-1α, as an oxygen-sensitive transcription factor, can regulate the expression of glycolysis genes in Th17 cells (111, 112). Blocking glycolysis will reduce the differentiation of TH17 cells, which facilitates the formation of Tregs (111). In addition, studies have shown that extracellular salts (like NaCl) (113, 114) and short-chain FA (115) affect the homeostasis of Th17 and Treg. This suggests that the metabolic microenvironment (i.e., nutrient and oxygen availability) may affect T cell polarization.

The differentiation of Th2 cells depends on the mTOR-mediated metabolic transition from OXPHOS to aerobic glycolysis, but the complete role of metabolism in Th2 polarization is not fully understood. Reprogramming of glycolysis and OXPHOS in Th2 cells depends on the activity of the small GTPase RhoA (46, 116). mTORC2 regulates RhoA activity and is essential for the production of Th2 cells (117). For the Th1 cell phenotype, mTORC1 may be essential (117). The transcription factor interferon regulatory factor 4 (IRF4) is also essential in the glycolytic reprogramming and differentiation of Th1 cells and can prevent the expression of glycolytic enzymes by regulating the transcription suppressor B-cell lymphoma 6 (BCL-6) (118).

The oxidation of free fatty acids (FFA) produces acetyl-CoA, which can be further metabolized in the TCA cycle, and FADH2 and NADH, which can be directly used by the electron transport chain (ETC) to produce ATP. Metformin is a metabolic stress factor that can activate the adenosine monophosphate-activated protein kinase (AMPK) energy sensor. It can enhance the production of CD8^+^ T cells (119). After AMPK is activated, it inhibits mTORC1 activity in response to energy stress (120). Consistent with this, rapamycin also inhibits mTORC1 and can enhance the production of CD8^+^ T cells (121, 122). On the other hand, mTORC2 is involved in FAO and stabilizes forkhead box protein O1 (FoxO1) through the Akt pathway to regulate the memory differentiation of CD8^+^ T cells (123, 124).

Treg cells depend on glycolysis for their differentiation and migration (125, 126). Glutamine or arginine can regulate the proliferation and activation of T cells and the expression level of Treg cell-specific transcription factor FoxP3 (107, 127).

These together reveal that a broad understanding of metabolic processes is emerging, which may help explain how metabolism determines the differentiation and function of Th1, Th2, Th17, Treg, and CD8^+^ T cells.

## The Influence of Other Immune Cells Metabolism Reprogramming on the Establishment of the Maternal-Fetal Interface

In addition to the above three types of immune cells, other immune cells with a smaller number also play an essential role in forming the maternal-fetal interface, such as dendritic cells (DC) and mast cells (MC).

DCs interact closely with other immune components and the endocrine system to maintain a pregnancy-friendly environment (128). As the most effective antigen-presenting cells in the immune system, DCs can regulate the differentiation of T cells (129, 130). In addition, they can inhibit the proliferation of NK cells (131) and may affect the polarization of macrophages (132). Abnormal DC activity is related to various pregnancy-related diseases, such as RIF (133), preterm birth (PTB) (134), PE (135), and perinatal cardiomyopathy (PPCM) (136), etc.

DCs are mainly divided into four subgroups, including conventional DCs (cDCs), plasmacytoid DCs (pDCs), Langerhans cells (LCs), and inflammatory monocyte-derived DCs (infDCs) (137). However, studies on immune changes in pregnancy mainly focus on cDC and pDC. Most current studies have shown that the cDC/pDC ratio of pregnant women is higher than that of non-pregnant women (134, 138), indicating that cDC may play an important role in the process of maternal-fetal tolerance. According to its maturity, DC can be divided into immature DC (imDC) and mature DC (mDC).

Decidual dendritic cells (dDCs) are a tolerant, immunomodulatory, and heterogeneous population, different from pDCs. dDCs are mainly immature DCs that express a combination of CD11c, HLA-G, CD14 and DC-SIGN (dendritic cell-specific ICAM-grabbing non integrin, CD209) (139). Observational studies have found that a higher level of mature peripheral or decidual DC is positively correlated with the development of adverse pregnancy (such as PE and abortion) (140, 141). This means that a delicate balance needs to be achieved between mature and immature dDC to ensure a successful pregnancy.

Metabolic processes have precise effects on DC functions, and manipulating these pathways can significantly change DC functions in specific ways. Due to the scarcity of DCs *in vivo*, *in vitro* experimental models are mainly used to study DC metabolism microenvironment.

A common phenomenon in immune cell activation, including DC, is the transition from catabolism, which is characterized by fatty acid oxidation and mitochondrial respiration, to anabolism. After immune cell activation, glycolysis is increased, and oxidative phosphorylation is decreased (142).

Under the stimulation of LPS, DCs can regulate mTOR signal, stabilize HIF1α and increase iNOS expression, induce the synthesis of endogenous NO. This process induces the synthesis of endogenous NO and inhibits the electron transport chain, thereby inhibiting mitochondrial oxidative phosphorylation, which increases cellular glycolysis to maintain intracellular ATP levels (143, 144). In fact, in addition to glucose, several amino acids also affect the mTORC1/HIF1α/iNOS signal, including leucine and glutamine, which are essential for mTORC1 activity, and arginine, which is the fuel for NO production on which iNOS relies (145). However, there are signs that once cells interact with T cells, the role of glucose in DCs initiating T cell responses will change. Activated T cells significantly increase the glucose and amino acid uptake rate, and the immediate microenvironment surrounding DC in the T cell cluster becomes deficient in nutrients. This competition for glucose or amino acids results in a prolonged T cell response (143).

The imDCs mainly rely on FAO for energy, which makes them have a longer lifespan (144). ω3 polyunsaturated fatty acids can inhibit the expression of the immune phenotype of DCs and the release of cytokines *in vitro*, and reduce their ability to stimulate T cell proliferation. The unsaturated fatty acid metabolite prostaglandin can regulate mDCs, upregulating the expression of costimulatory molecules and pro-inflammatory cytokines at low concentrations while inhibiting the expression at higher concentrations (5). However, most of these data come from In vitro studies, the metabolic environment in which DC competes with nearby cells for nutrition is difficult to imitate *in vitro*, and it is also difficult to measure *in vivo*. However, it is undeniable that the metabolic reprogramming of DC is of vital importance to its function.

Mast cells also have a positive effect on embryo implantation (146). It can secrete hemcryptin-1 (a glycan-binding protein) to support the survival of trophoblasts, placenta and fetal growth. Mice lacking mast cells (including uterine mast cells) have impaired implantation (147). Uterine NKs and MCs can also balance their influence at the fetal-maternal interface and jointly promote SA remodeling and fetal position (148). However, no researchers have conducted metabolic reprogramming studies on MCs, so this review will not describe it a lot.

## Summary and Outlook

**Figure 3:**
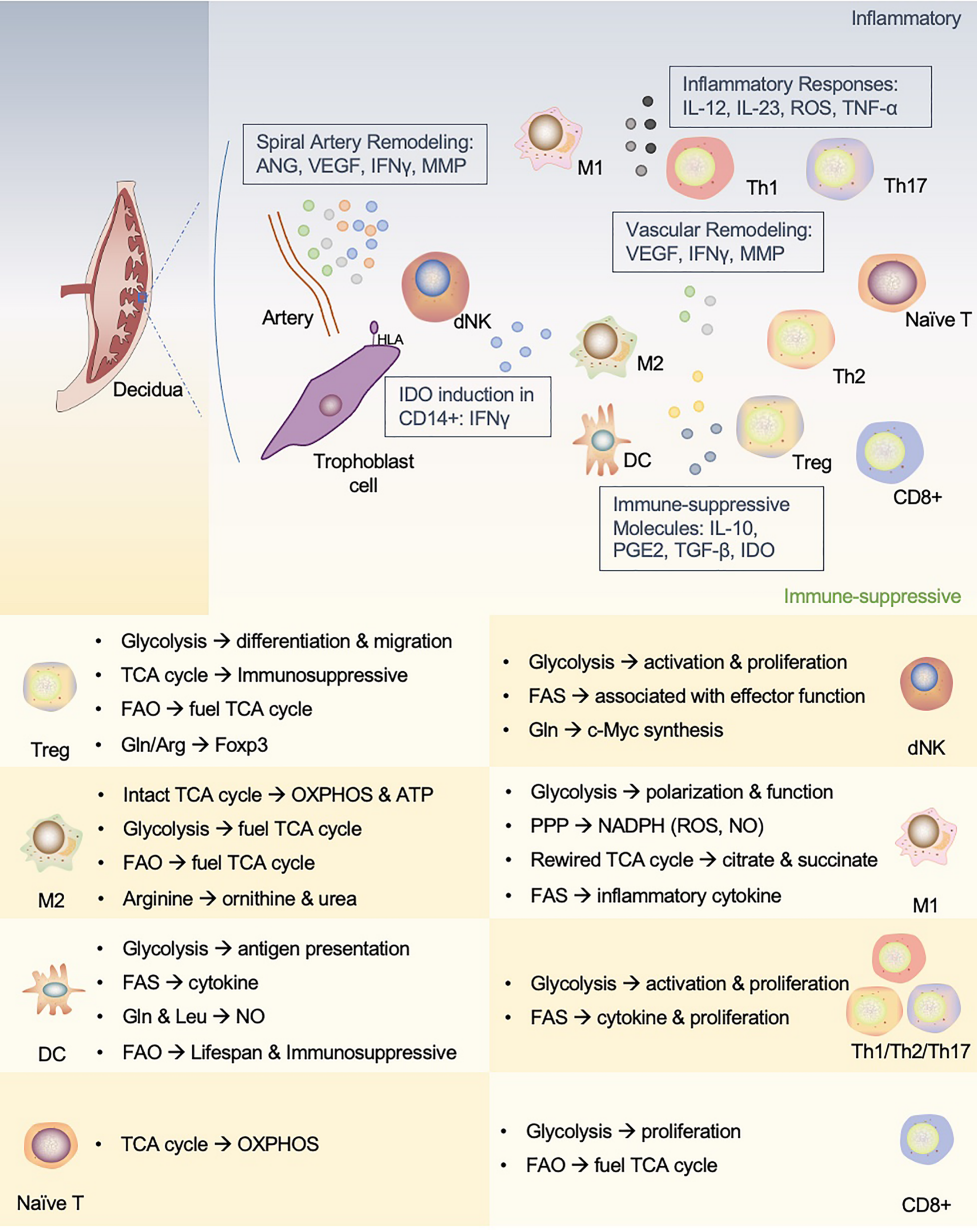
An interactive network of immune cells and underlying metabolic phenotypes at the maternal-fetal interface. This figure proposes a unique immune microenvironment at the maternal-fetal interface and potential metabolic mechanisms that support phenotypic adaptation during pregnancy. In human maternal-fetal interface, trained NK cells promote inflammatory and pro-angiogenic cytokines that regulate angiogenesis and trophoblast invasion and migration. The release of IFN-γ also upregulates IDO expression in antigen-presenting cells (APCs, such as macrophages and DCs), which influences T cell activation and facilitates Treg cell induction. In addition, APCs play a role in vascular remodeling in early pregnancy. dNK, decidual NK cells; M1, M1 macrophages; M2, M2 macrophages; Th1, T-helper 1 cells; Th2, T-helper 2 cells; Th17, T-helper 17 cells; Treg, regulatory T cells; DC, dendritic cell; ANG, angiotensin; VEGF, vascular endothelial growth factor; MMP, matrix metalloprotein; IDO, indoleamine2,3-dioxygenase; PGE2, prostaglandin E2.

Immune homeostasis relies on immune cells to acquire appropriate functions by adaptively regulating their metabolic preferences to rebalance their immune microenvironment. This review summarized the metabolic phenotypes of the main immune cells in the context of maternal-fetal interface (**Figure 3**). Investigation on the metabolic reprogramming of immune cells at the maternal-fetal interface can help us understand the mechanisms of pregnancy and provide insights into developing diagnostic and therapeutic approaches for pregnancy-related diseases. However, research on immunometabolism in pregnancy is still lagging due to clinical ethics and technological developments.

First of all, in most cases, researchers only focus on the most abundant cell groups found in the blood or bone marrow rather than tissue residents or recruited cells that actively participate in host defense and tissue homeostasis. Secondly, the current research does not consider the observed diversity of immune cell lineages or tissue-specific functions. They only consider the metabolic reprogramming of a single or a group of immune cells, ignored interactions between various immune cells in the tissue that may cause metabolic reprogramming again. Thirdly, although many inducers and sensors have been identified for activating immune cells, we lack an understanding of how these signals are integrated into cohesive metabolic programs that support cell effector functions. Last but not the least, it is technically challenging to identify the relative quantity of “all metabolites” in metabolomics. For example, GC-MS effectively quantifies organic acids (such as TCA cycle intermediates), but it is not practical for most glycolysis intermediates. Some volatile or unstable substances may not be detected by the LC-MS method, while GC-MS may be a better choice. Thus, multiple platforms need to be used to generate a complete metabolic data set.

As the cost of applying these new techniques decreases and the application becomes more comprehensive, more valuable, original breakthroughs can be made in this field. There are many questions urgently requiring in-depth investigation and deliberation regarding reproductive immunology. Such as which side plays the dominant role in establishing the maternal-fetal immune interface during gestation establishment? How does the maternal-fetal immune system initiate immune tolerance to the exemption of the embryo from invasive behavior? What methods can be used to detect the temporal and spatial tipping points at which immune tolerance occurs to determine where and when to take the appropriate clinical decisions to prevent the risk? Therefore, the development of new tools and experimental models is imperative. We can be confident that researchers will make considerable progress in understanding reproductive, immune metabolism shortly.

## Author Contributions

YW, TY, and LD conceived the original idea and the structure of the manuscript. YW, JD, and JL drafted the first version of the manuscript. YW and SC developed the figures. SL and LH critically provided critical feedback and helped shape the manuscript. LD, TY, and YZ supervised and revised the manuscript. All authors contributed to the article and approved the submitted version.

## Funding

This work was supported by the following grants: the National Key Research and Development Program of China (No. 2018YFC1004601, 2018YFC1003900/04), and the National Natural Science Foundation of China (No. 81801540), and Shenzhen Natural Science Foundation (JCYJ20190813161801676).

## Conflict of Interest

Authors SL, LH and LD were employed by company Shenzhen Jinxin Medical Technology Innovation Center, Co., Ltd.

The remaining authors declare that the research was conducted in the absence of any commercial or financial relationships that could be construed as a potential conflict of interest.

## Publisher’s Note

All claims expressed in this article are solely those of the authors and do not necessarily represent those of their affiliated organizations, or those of the publisher, the editors and the reviewers. Any product that may be evaluated in this article, or claim that may be made by its manufacturer, is not guaranteed or endorsed by the publisher.
